# *De Novo* Sequencing and Characterization of the Transcriptome of Dwarf Polish Wheat (*Triticum polonicum *L.)

**DOI:** 10.1155/2016/5781412

**Published:** 2016-06-27

**Authors:** Yi Wang, Chao Wang, Xiaolu Wang, Fan Peng, Ruijiao Wang, Yulin Jiang, Jian Zeng, Xing Fan, Houyang Kang, Lina Sha, Haiqin Zhang, Xue Xiao, Yonghong Zhou

**Affiliations:** ^1^Triticeae Research Institute, Sichuan Agricultural University, Wenjiang, Sichuan 611130, China; ^2^College of Resources, Sichuan Agricultural University, Wenjiang, Sichuan 611130, China

## Abstract

Construction as well as characterization of a polish wheat transcriptome is a crucial step to study useful traits of polish wheat. In this study, a transcriptome, including 76,014 unigenes, was assembled from dwarf polish wheat (DPW) roots, stems, and leaves using the software of Trinity. Among these unigenes, 61,748 (81.23%) unigenes were functionally annotated in public databases and classified into differentially functional types. Aligning this transcriptome against draft wheat genome released by the International Wheat Genome Sequencing Consortium (IWGSC), 57,331 (75.42%) unigenes, including 26,122 AB-specific and 2,622 D-specific unigenes, were mapped on A, B, and/or D genomes. Compared with the transcriptome of *T. turgidum*, 56,343 unigenes were matched with 103,327 unigenes of* T. turgidum*. Compared with the genomes of rice and barley, 14,404 and 7,007 unigenes were matched with 14,608 genes of barley and 7,708 genes of rice, respectively. On the other hand, 2,148, 1,611, and 2,707 unigenes were expressed specifically in roots, stems, and leaves, respectively. Finally, 5,531 SSR sequences were observed from 4,531 unigenes, and 518 primer pairs were designed.

## 1. Introduction

Due to the high-thousand kernel weight, elongated and plump kernels, high Zn, Fe, and Cu concentrations in seeds [1], high amylose content in seeds [2], and alternatively dwarfing genes [3, 4], polish wheat (2*n* = 4*x* = 28, AABB,* Triticum polonicum* L.) attracts the interest of producers and breeders [1]. However, polish wheat may be a hybrid of* Triticum ispahanicum* H. and* T. durum* (2*n* = 4*x* = 28, AABB) [5, 6]. The genetic background of polish wheat, especial Chinese polish wheat, is low similarity with* T. durum*,* T. turgidum* (2*n* = 4*x* = 28, AABB), and* T. aestivum* (2*n* = 6*x* = 42, AABBDD) [7, 8]. It is therefore inappropriate to reveal the genetic information of polish wheat using the genome or transcriptomes of* T. turgidum* and* T. aestivum* [9–12].

With advances in next-generation sequencing technology, RNA sequencing (RNA-Seq), with high throughput, produced sequences and then mapped them on a reference genome, or* de novo* assembles a better depiction of transcriptome [9, 10, 13–15] and has been/is being widely used in model organisms and nonmodel organisms to study biological processes and applications, such as SNP and gene discovery, SSR mining, and identification of differentially expressed genes [15–17]. Although the draft genome and transcriptome of* T. aestivum* and the transcriptome of tetraploid wheat were released [9–12], transcriptome information of polish wheat is not constructed and reported. Construction as well as characterization of a polish wheat transcriptome, therefore, is a crucial step to study useful traits in polish wheat.

Dwarf polish wheat (DPW) with a recessive dwarfing gene [3] was originally collected from Tulufan, Xinjiang province, China. Therefore, the genetic similarity between DPW and* T. durum*,* T. turgidum*, and* T. aestivum* should be low [7, 8]. In this study, the transcriptome of DPW was constructed and characterized. Additionally, the transcriptome was compared with the genomes of barley, rice, and comment wheat and the transcriptome of* T. turgidum*. Finally, some SSR markers were mined.

## 2. Materials and Methods

### 2.1. Raw Reads

10 DPW raw reads databases contained 697.13 million 100 bp paired-end raw reads that were downloaded from the NCBI sequence read archive (SRA) database. Among these raw reads databases, 370.82, 115.51, and 210.80 million reads were generated from roots (SRA numbers: SRR2973581, SRR2973582, SRR2973583, and SRR2973584; unpublished data), stems (SRA numbers: SRR2969441 and SRR2969444; [18]), and leaves (SRA numbers: SRR2973592, SRR2973593, SRR2973594, and SRR2973595; unpublished data), respectively. Roots (four samples) were collected from seedlings; stems (two samples; [18]) and leaves (four samples) were collected at the booting stage. All these 10 samples were sequenced by our laboratory using the 100 bp protocol on Illumina Hiseq 2000 platform. All sequenced information was briefly described as Wang et al. [18].

### 2.2. Transcriptome Assembly and CDS (Coding Sequence) Prediction

Reads containing adapters, poly-N, and low quality reads were removed using Novogene-written perl scripts to produce clean reads. Meanwhile, GC content and sequence duplication level of the clean data were calculated. All unigenes were assembled using the software of Trinity (V2012-10-15) [19] with minimum *K*-mer coverage of 2, and other parameters were default. Unigenes were defined using the methods of Zhang et al. [14] and Krasileva et al. [10].

### 2.3. Gene Functional Annotation

The functions of unigene were annotated using a series of databases, including blastx against the NCBI nonredundant protein (Nr), NCBI nucleotide collection (Nt) and Swiss-Prot databases with 10^−5^ as an *e*-value cutoff, and hmmscan against protein family (Pfam). Functional categories of unigenes were grouped using Kyoto Encyclopedia of Genes and Genomes (KEGG, http://www.genome.jp/kegg/), Clusters of Orthologous Groups of Proteins database (KOG/COG, http://www.ncbi.nlm.nih.gov/COG/), and Gene Ontology ([20]; http://www.geneontology.org), respectively.

### 2.4. Tissue-Specific Expression Analysis

Clean reads were aligned against assembled transcriptome to produce read count using the package of RSEM [21]. The read count of each unigene was converted into RPKM values for normalizing gene expression using the RPKM method [13]. If the value of RPKM was 0 (N/A), the unigene was not expressed. Tissue-specific unigenes were selected out according to RPKM values of unigenes among roots, leaves, and stems.

### 2.5. Comparative Genomics Analysis

All unigenes were blasted against draft wheat genome [22] with *e*-value < 10^−5^, coverage > 90%, and alignment length > 200 bp. All unigenes were also blasted against the transcriptome of* T. turgidum* [10] with *e*-value < 10^−5^.

Peptide sequences of barley were obtained from the website http://plants.ensembl.org/hordeum_vulgare/Info/Index [23], and peptide sequences of rice were obtained from the website http://plants.ensembl.org/Oryza_sativa/Info/Index [24]. Sequence alignments were performed using blastx with *e*-value < 10^−5^, alignment length > 100, and identity > 80%.

### 2.6. SSR Mining and Primer Design

SSR sequences (SSRs) were observed using the software of MIcroSAtellite (MISA, http://pgrc.ipk-gatersleben.de/misa/) as described by Zhang et al. [15]. The SSRs were considered to contain motifs with one to six nucleotides in size and a minimum of 5 contiguous repeat units. Based on these SSRs, primers were designed using the software of Primer 3.

## 3. Results and Discussion

### 3.1. Sequencing and De Novo Assembly of the DPW Transcriptome

Although 697.13 million (370.82 in roots, 115.51 in stems, and 210.80 in leaves) 100 bp paired-end raw reads were generated from DPW, after cleaning and quality checks, 671.49 million (361.96 in roots, 108.11 in stems, and 201.32 in leaves) 100 bp paired-end clean reads were used for assembly. Finally, 76,014 unigenes (lengths of unigenes ranged from 201 to 19,201 bp) with mean sizes of 872 bp (Table 1, all assembled unigenes have been deposited at GenBank under the accession GEDT00000000) were assembled. The number of unigenes in this transcriptome was less than the transcriptome of* T. turgidum* which contained 140,118 unigenes with mean sizes of 1,299 bp [10] but was more than the transcriptome of* T. turgidum* cv. Langdon that contained 40,349 unigenes [17].

### 3.2. Functional Annotation of Unigenes

Among these 76,014 unigenes, 61,748 (81.23%) unigenes were functionally annotated in at least one database of the NCBI Nr, Nt, Swiss-Prot, KEGG, KOG, and COG using blastx with an *e*-value below *e* ^−5^ (the GenBank accession GEDT00000000). Of the 61,748 annotated unigenes, 11,207 (18.15%), 28,104 (45.51%), 6,830 (11.06%), 17,877 (28.95%), 22,930 (37.13%), 44,878 (72.68%), and 58,659 (95.00%) unigenes were classified into 26 COG categories, three GO functional categories [molecular function (15,684), biological process (4,637), and cellular components (7,783)], KEGG, KOG, pfam, Nr, and Nt, respectively. All annotated information was also deposited at GenBank under the accession GEDT00000000.

Previously well-studied transcriptomes reported that many unigenes were not functionally annotated, such as 30% in* T. turgidum* [10], 32.12% in peanut [15], and 45.10% in* Dendrocalamus latiflorus* [14]. In this study, 14,266 (23.10%) unigenes were not functionally annotated in any database. As proposed by Krasileva et al. [10], these unigenes might be (1) wheat-specific genes or highly divergent genes; (2) expressed pseudogenes; (3) noncoding transcribed sequences; (4) pieces of 5′ and 3′ UTRs; and (5) general assembly artifacts. Absolutely, some of these unannotated unigenes, such as noncoding transcribed RNAs, also regulate various cellular processes or other regulations in wheat [25].

**Figure 1:**
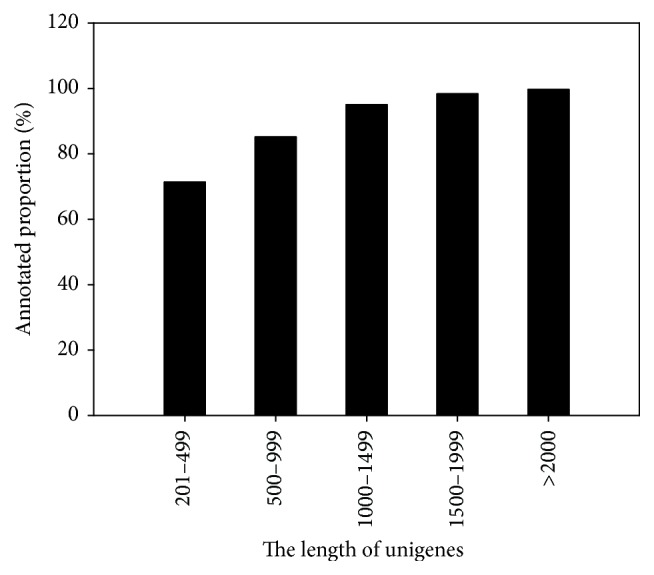
Annotated proportion of DPW transcriptome with different lengths.

On the other hand, as the lengths of unigenes were longer, the annotated efficiencies were higher [14]. In the present study, 99.67% of unigenes with more than 2,000 bp, 98.34% of unigenes with 1,500–1,999 bp, and 95.02% of unigenes with 1,000–1,499 bp were annotated in at least one public database. However, 85.08% of unigenes with 500–999 bp and 71.39% of unigenes with 201–499 bp were annotated (Figure 1).

### 3.3. Comparison with the Genomes or Transcriptome of Wheat,* T. turgidum*, Barley, and Rice

**Figure 2:**
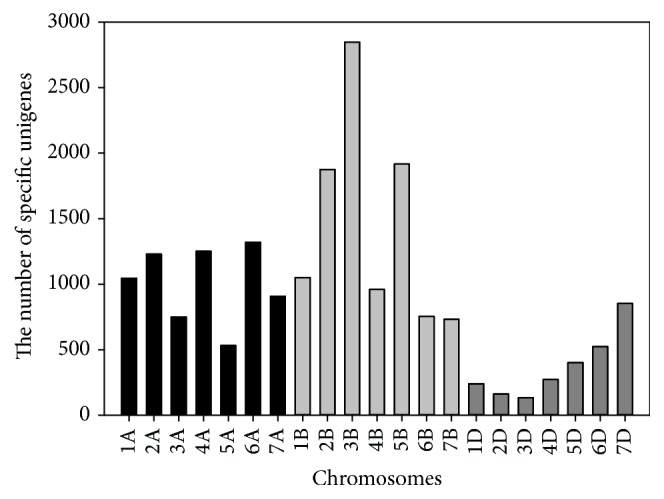
The number of unigenes mapped specifically on different wheat chromosomes.

Blasted against the draft wheat genome released by IWGSC, 57,331 (75.42%) unigenes were mapped on A, B, and/or D genomes, including 26,122 AB genome-specific and 2,622 D genome-specific unigenes, respectively (SFile 1, in Supplementary Material available online at http://dx.doi.org/10.1155/2016/5781412; Figure 2). Among 26,122 A/B genome-specific unigenes, 7,785 and 11,291 unigenes were mapped specifically on A and B genomes, respectively (Figure 2). Meanwhile, all unigenes were compared with the transcriptome of* T. turgidum* [10]. 56,343 (74.12%) unigenes were successfully matched with 103,327 (73.74%) unigenes of* T. turgidum* (SFile 2). Approximately, 25% of unigenes of DPW transcriptome did not match on draft wheat genome or the transcriptome of* T. turgidum*, which suggested polish wheat has low genetic similarity with* T. durum*,* T. turgidum,* and* T. aestivum* [7, 8] or different tissues for constructing transcriptomes might product some tissue-specific unigenes [10, 11]. Interestingly, 2,622 unigenes were mapped specifically on D genome (Figure 2, SFile 1). Meanwhile, polish wheat may be a hybrid of* T. ispahanicum* and* T. durum* [5, 6]. This result indicated that AB genomes might give rise to the D genome through homoploid hybrid speciation [26].

Meanwhile, all unigenes were also blasted against the published genomes of barley [23] and rice [24] with an *e*-value below *e* ^−5^ and more than 100 matched amino acids. 14,404 (18.95%, SFile 3) and 7,007 (9.21%, SFile 4) unigenes were matched with 14,608 genes of barley and 7,708 genes of rice, respectively, which were lower than 70% of unigenes of bread wheat matched with rice and barley genes [11].

### 3.4. Tissue-Specific Unigenes

**Figure 3:**
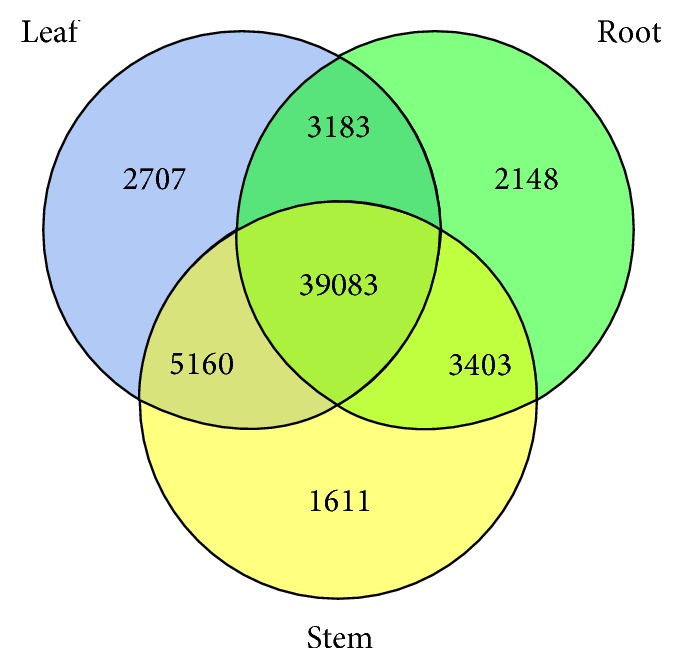
The number of unigenes expressed in stems, leaves, and roots, respectively.

Since this transcriptome was constructed from roots, leaves, and stems, there should be some tissue-specific unigenes. Among 76,014 unigenes, 39,083 unigenes, which were involved in basic development and life cycles, such as translation, secondary metabolites biosynthesis, DNA replication, recombination and repair, transcription, signal transduction, carbohydrate transport and metabolism, cell cycle control, cell division, chromosome partitioning, chromatin structure and dynamics, coenzyme transport and metabolism, defense mechanisms, energy production and conversion, and RNA processing and modification, coexisted in all tissues (Figure 3, SFile 5). 5,160, 3403, and 3183 unigenes coexisted in leaves and stems, roots and stems, and leaves and roots, respectively (Figure 3, SFile 5).

On the other hand, 2,148 unigenes, such as* ABC transporter B and C members*,* high affinity nitrate transporters*,* peroxidases,* and* glutathione S-transferases* which participated in metal tolerances [27–30], were specifically expressed in roots treated with Cd and Zn (Figure 3, SFile 5). 1611 unigenes, such as some* cytochrome P450*,* ABC transporter B and G members*,* beta-galactosidases*,* glucoside dioxygenases*, a*uxin efflux carriers,* and* glycosyltransferases *that participated in phytohormones transport, cell wall metabolism [31–33], respectively, were stem-specific unigenes (Figure 3, SFile 5). 2,707 unigenes, such as* G-type lectin S-receptor-like serine* and* leucine-rich repeat receptor-like protein kinase* which were involved in abiotic-stresses tolerance [34–36], were leaf-specific unigenes (Figure 3, SFile 5).

### 3.5. SSR Mining

**Figure 4:**
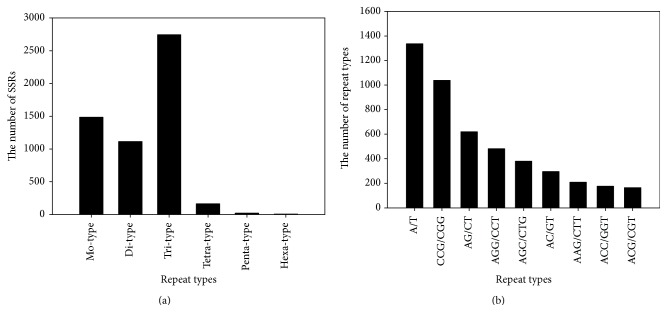
Characterization of SSRs mining. (a) The number of SSRs classified into different repeat types; (b) the number of abundant repeat types.

Due to high level of polymorphism, locus specificity, codominance, convenience, and uniform distribution throughout the genome [8], SSR markers have been/are being used in various studies in wheat [8, 37]. In the present study, 5,531 SSRs were observed from 4,531 unigenes with more than 1000 bp. Of them, 810 unigenes contained more than 1 SSR; 241 SSRs were compound formation (SFile 6). These SSRs included 1,485 (26.85%) mono-nucleotide motifs, 1,113 (20.12%) di-nucleotide motifs, 2,744 (49.61%) tri-nucleotide motifs, 163 (2.95) tetra-nucleotide motifs, 19 (0.34%) penta-nucleotide motifs, and 7 (0.13%) hexa-nucleotide motifs (Figure 4(a)). The most abundant repeat type was A/T, followed by CCG/CGG, AG/CT, AGG/CCT, AGC/CTG, AC/GT, AAG/CTT, ACC/GGT, and ACG/CGT, respectively (Figure 4(b)). Based on these 5531 SSRs, 4518 primer pairs were designed using the software of Primer 3 (SFile 7).

## Supplementary Material

SFile 1 The location of unigenes in wheat chromosomes. SFile 2 Comparative information against *T. turgidum*. SFile 3 Comparative information against barley. SFile 4 Comparative information against rice. SFile 4 The information of unigenes expressions. SFile 6 The sequences of SSRs in unigenes. SFile 7 The primers of SSRs.

## Figures and Tables

**Table 1:** The information of transcriptome.

	Number
100 bp paired-end raw reads (million)	697.13
100 bp paired-end clean reads (million)	671.49
Unigenes	76,014
Mean unigenes size	866 bp
Min unigenes size	201 bp
Max unigenes size	19,201 bp
Unigenes mapping to AABBDD genome	57,311
Unigenes unmapping to AABBDD genome	18,683
Unigenes mapping to AABB transcriptome of *T. turgidum*	56,343
Unigenes unmapping to AABB transcriptome of *T. turgidum*	19,671
Annotated unigenes	61,748
Unannotated unigenes	14,266
